# Edema and Subjective Discomfort After Barbed Stayed Bridge Pharyngoplasty (BSBP)

**DOI:** 10.3390/jcm14207402

**Published:** 2025-10-20

**Authors:** Annalisa Pace, Giannicola Iannella, Antonino Maniaci, Salvatore Cocuzza, Antonio Moffa, Danilo Alunni Fegatelli, Alessandra Manno, Armando De Virgilio, Manuele Casale, Giuseppe Magliulo

**Affiliations:** 1 Department of ‘Organi di Senso’, University “Sapienza”, Viale dell’Università 33, 00185 Rome, Italy; giannicola.iannella@uniroma1.it (G.I.); alessandra.manno@uniroma1.it (A.M.); armando.devirgilio@uniroma1.it (A.D.V.); giuseppe.magliulo@uniroma1.it (G.M.); 2 Department of Medicine and Surgery, University of Enna “Kore”, 94100 Enna, Italy; tn.maniaci92@gmail.com; 3 Department of Medical and Surgical Sciences and Advanced Technologies “GF Ingrassia”, ENT Section, University of Catania, Via S. Sofia 78, 95125 Catania, Italy; s.cocuzza@unict.it; 4 Integrated Therapies in Otolaryngology, Campus Bio-Medico University Hospital Foundation, Via Alvaro del Portillo, 00128 Rome, Italy; a.moffa@unicampus.it (A.M.); m.casale@unicampus.it (M.C.); 5 Department of Life Sciences, Health and Health Professions, Link Campus University, 00165 Rome, Italy; d.alunnifegatelli@unilink.it

**Keywords:** obstructive sleep apnea, oropharynx, surgical treatment of obstructive sleep apnea

## Abstract

**Background**: Obstructive Sleep Apnea (OSA) is commonly treated with CPAP, though low patient compliance often limits its long-term use. Surgical alternatives, such as Barbed Pharyngoplasty, have been developed to address retro-velar collapse. A recent technique, Barbed Stayed Bridge Pharyngoplasty (BSBP), aims to preserve oropharyngeal anatomy while enhancing airway stability. This study evaluates the immediate postoperative outcomes and patient discomfort following BSBP for OSA. **Material and Method**: Thirty patients (mean age 40.7 ± 8.9 years; BMI 25.9 ± 1.7) underwent BSBP at Sapienza University of Rome between January 2022 and January 2024. Inclusion criteria included AHI 15–30, BMI ≤ 35, and specific DISE findings. Postoperative outcomes were evaluated using polysomnographic data (AHI, ODI), the Epworth Sleepiness Scale (ESS), pain scores (VAS), edema grading (Ezzat score), and the PPOPS questionnaire. Follow-ups were performed at 12, 24, and 48 h; 1 month; and 6 months postoperatively. **Results**: Thirty patients (average age 40.7) with mild to moderate OSA underwent surgery. After six months, there was a significant reduction in AHI (from 23.4 ± 2.1 to 7.2 ± 2.6) and ODI (from 21.0 ± 4.1 to 6.5 ± 2.1), along with a statistically significant improvement in the Epworth Sleepiness Scale. Postoperative pain (VAS) decreased from 3.0 ± 1.5 at 12 h to 0.2 ± 0.5 at one month, and edema (Ezzat score) decreased from 2.0 ± 0.6 at 12 h to 0.0 ± 0.0 at one month, both with significant differences. The PPOPS questionnaire scores remained relatively stable, with mean values of 4.9 ± 2.3 at 12 h, 2.7 ± 1.6 at 24 h, 1.2 ± 1.1 at 48 h, and 0.5 ± 0.7 at one month, showing statistically significant change (*p* < 0.005). No postoperative bleeding occurred. According to Sher’s criteria, the procedure was consistently effective. **Conclusions**: BSBP significantly reduces AHI and ODI, demonstrating effective symptom resolution with minimal discomfort and rapid recovery. These results suggest that BSBP may be a viable, less invasive surgical technique for OSA surgeries.

## 1. Introduction

Obstructive sleep apnea (OSA) is a condition that typically employs CPAP as first-line treatment [1]. However, since CPAP is not well-tolerated, velopharyngeal surgery may represent a valid therapeutic alternative for OSA patients with retro-velar and/or oropharyngeal collapse [2].

The history of OSA surgery can be identified with an initial “demolitive” era of palatal surgery, followed by a gradual shift towards a “mini-invasive” attitude based on the use of barbed sutures [3,4].

One of the most widely performed barbed techniques is Barbed Reposition Pharyngoplasty (BRP), proposed by Vicini et al. [5]. BRP stiffens the pharyngeal lateral wall using barbed sutures, increasing the cross-section of the retropalatal area and oropharyngeal inlet. Notwithstanding this, the technique is termed mini-invasive. Still, some demolition steps are required: tonsillectomy, removal of the “fat pad” area, and partial sectioning of the palatopharyngeal muscles (PPM) in their inferior portion [6].

Magliulo et al. recently proposed a new technique called Barbed Stayed Bridge Pharyngoplasty (BSBP) [7]. This surgical technique compares the soft palate to a cable-stayed bridge, improving the system’s dynamic stability while avoiding demolition steps other than tonsillectomy. Hence, this technique is less traumatic than BRP since it respects oropharyngeal anatomy and physiology.

It is important to remember that the soft palate is a dynamic structure in which each muscle has a particular action and histology [8]. Therefore, transcut PPM may produce fibrosis that reduces the system’s control and normal muscle equilibrium. Conversely, it is fundamental to reach the same goal of BRP: increasing the system’s stiffness and enlarging the oropharyngeal space. Therefore, BSBP starts reinforcing PPM with a barbed suture, modifying its position from posterior to lateral. This way, the PPM’s not transcut has a strength used to control the enlargement by barbed sutures. Moreover, the absence of fibrosis may increase the system’s elasticity.

After one year of follow-up, polysomnographic results of BSBP showed good outcomes and resolved the OSA condition [7].

The primary objective of this study was to evaluate the immediate postoperative clinical outcomes and discomfort of the BSBP technique. Moreover, the secondary objective was to assess the initial efficacy of six months regarding polysomnographic findings.

## 2. Materials and Methods

This study was performed at the Sense of Organ Department of Sapienza University of Rome between January 2022 and January 2024. It was approved by the local Sapienza University Ethical Committee (RIF: 6267), following the principles of the Declaration of Helsinki. Each patient enrolled in the study signed an informed consent.

**Figure 1:**
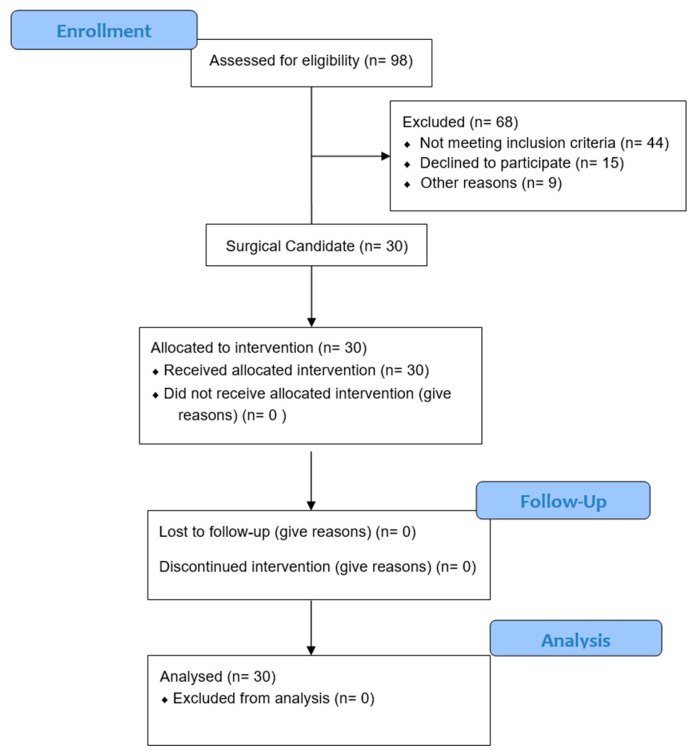
CONSORT patient flow diagram.

CONSORT patient flow diagram Figure 1.

All enrolled patients performed the Epworth Sleepiness Scale with results ranging from 0 to 24. Results from 0 to 10 show average (normal) daytime sleepiness; a result from 11 to 24 indicates excessive (abnormal) daytime sleepiness. All patients underwent home sleep apnea testing (HSAT) type III to define the severity score on the Apnea-Hypopnea Index (AHI) based on the AASM classification 2017 [9].

The patients were classified using the Friedman staging system and the Lingual Tonsil Hypertrophy grading system [10].

Everyone underwent Drug-Induced Sleep Endoscopy (DISE) and VOTE (Velum, Oropharynx, Tongue, Epiglottis) classification [11].

### 2.1. Inclusion Criteria

- Age between 30 and 60.
- BMI ≤ 35 kg/m^2^.
- Moderate OSA patients with AHI > 15 and < 30.
- V1 or 2 concentric/lateral–lateral; O0 or 1; T0; E0.
- Friedman Tongue grade I–II–III.
- Lingual tonsil hypertrophy > 1.

### 2.2. Exclusion Criteria

- Age < 30 and > 60.
- BMI < 35 kg/m^2^.
- AHI > 15/h and > 30 h.
- VOTE is different from V1 or 2 concentric/laterolateral; O0 or 1; T0; E0.
- Patients who need a multilevel approach.
- Friedman Tongue grade IV.
- Lingual tonsil hypertrophy > I.

### 2.3. Surgical Technique: Barbed Stayed Bridge Pharyngoplasty (BSBP)

Barbed stayed bridge pharyngoplasty (Video S1) is performed under general anesthesia, using orotracheal intubation and an armored tube. The patient is supine with his head extended (pillow under the shoulders). A David mouth gag was placed. The first step is bilateral tonsillectomy, preserving the palatopharyngeal muscle (PPM) and the palatoglossal muscle (PGM). The muscles are identified and unstuck without any transection. Landmarks are placed using a pen: posterior nasal spine in the midline; pterygomandibular raphe divided into three points: superior, middle, and inferior; the intermediate point is located a little bit down along a parasagittal line drawn strictly between the nasal spine and raphe.

The needle starts from the posterior nasal spine and is continued laterally to an intermediate point. The needle tip is extracted from the pterygomandibular raphe. The needle reaches the tonsillar fossa and crosses the entire PPM to reach its inferior margin. The needle is introduced to roll up from lateral to medial, the posterior margin of the PPM bundle, without involving the PGM. This is a key point in reinforcing the muscle and transposing the PPM from a posterior position to a lateral one in the oropharynx.

The needle is pulled close to the bare tonsillar fossa’s medial limit at the transition line between the muscle and the intact pharyngeal mucosa. Then, after two rotations, the needle is extracted and transposed to the inferior part of the raphe. From the same point, it returns to the lateral inferior part of the PPM, where it rises in the middle part, repeating the same operation. Finally, it is also completed in the superior portion. Finally, needle passage is performed back lateral to the raphe, applying the proper tension to the suture to reposition the palatopharyngeal muscle more laterally and anteriorly and splint and tighten the lateral wall.

### 2.4. Post-Surgery Evaluation

Hospitalization had a duration of 2 days. Postoperative drugs included prednisolone 20 mg intravenous (IV) 2 times a day for the first 2 days and paracetamol 1 g IV only for the first day; opioids were not administered.

ENT examination was performed at 12, 24, and 48 h, and then after 1 month (Video S2), and pain was evaluated using a Visual Analogical Scale (VAS) from 0 to 10 [12,13].

At any time, the edema scale designed by Ezzat et al. [14] was performed. Edema was classified as follows: grade 0: no edema, grade 1: local surface area edema ≥ 1 cm^2^, grade 2: 1–2 cm^2^, grade 3: 2–4 cm^2^, grade 4: ≥4 cm^2^.

At each assessment, patients filled out the PPOSP questionnaire (Palatal Post-Operative Problems Score) [15,16] (Table 1). This is based on 12 questions that test swallowing problems after surgery, nasal voice, weight loss, swallowing pain or discomfort, and eventual discouragement of the procedure. Each answer is scored from 0 (best results) to 3 (worst results). The total score ranges from 0 to 36.

At six months, it was linked to the HSAT. We considered it a success according to Sher’s definition, which consisted of reducing the postoperative AHI below 20/h.

### 2.5. Statistical Analysis

Descriptive statistics were used to summarize the characteristics of the study sample: numerical data were reported as mean values with standard deviation (SD) and median with interquartile range (IQR). Statistical comparisons were conducted using the Wilcoxon rank-sum test for numerical variables and Fisher’s exact test for categorical binary variables. Statistical significance was set at 0.05. All the analyses were performed using the statistical software R (version 4.0.4).

## 3. Results

Thirty patients (pts) were enrolled (age = 40.7 ± 8.9 years; BMI =25.9 ± 1.7; 18 males and 12 females) with the following Friedman staging system: 6 pts stage I, 21 pts stage II, 2 pts stage III; lingual tonsil hypertrophy grading system: 24 pts grade 0, 6 pts grade I.

At VOTE classification there were 5 pts with V2ccO1T0E0; 7 pts V2ll=1T0E0; 5 pts V2llO0T0E0; 2 pts V2ccO0T0E0; 3 pts V1llO1T0E0; 1 pts V1ccO0T0E0; 3 pts V1llO0E0T0; 4 pts V1ccO0T0E0.

The mean preoperative AHI value was 23.4 ± 2.1, and the ODI was 21.0 ± 4.1. At six months, the postoperative values were AHI = 7.2 ± 2.6 and ODI = 6.5 ± 2.1.

The comparison between AHI values preoperatively and postoperatively at six months always showed a statistical difference (*p* < 0.05) (Table 1). Similar findings were observed for ODI comparison with a *p* < 0.05 (Table 1), while t90% was not statistically significant (*p* > 0.05). Epworth Sleepiness scale between pre-surgery and after 6 months showed a statistical difference (Table 1). Moreover, according to Sher’s definition, there was always success.

The violin boxplot in Figure 1 represents the difference between AHI, ODI, and ESS before and after six months of treatment.

**Figure 2:**
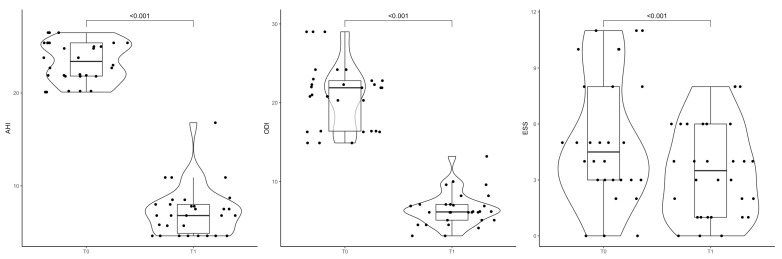
The violin boxplot shows the difference between AHI, ODI, and ESS pre-surgery and after six months.

The mean VAS values for pain were 3.0 ± 1.5 at 12 h, 2.5 ± 1.2 at 24 h, 2.4 ± 0.9 at 48 h, 0.2 ± 0.5 at 1 month (Table 2). The differences were statistically significant when comparing 12 h, 24 h, and 48 h with 1 month (Table 2; Figure 2).

**Figure 3:**
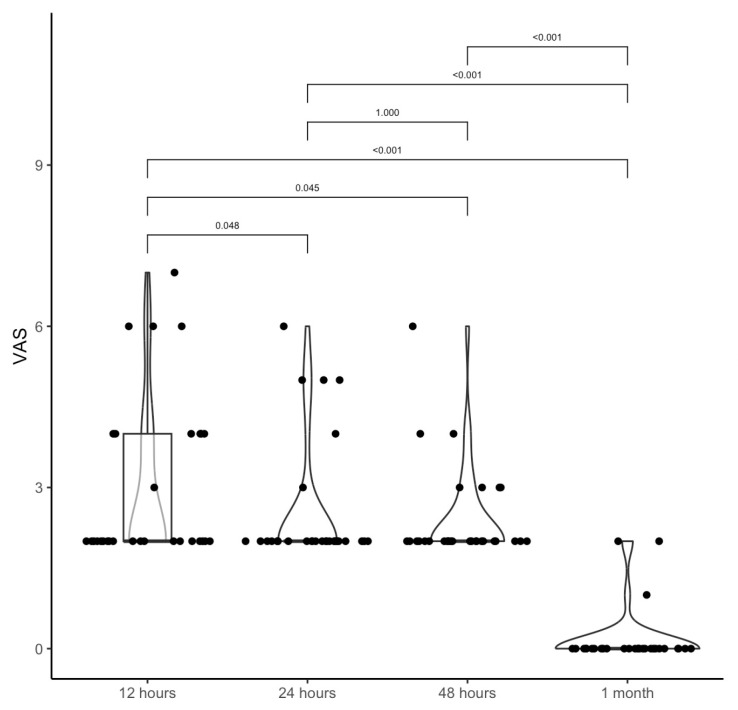
Violin boxplot shows the difference between VAS pre-surgery and 12 h, 24 h, 48 h after surgery, and after 1 month.

The mean values of Edema calculated with Ezzat score were 2.0 ± 0.6 at 12 h; 1.6 ± 0.7 at 24 h; 1.4 ± 0.7 at 48 h; 0.0 ± 0.0 at 1 month (Table 3). The differences were statistically significant when comparing 12 h, 24 h, and 48 h with 1 month (Table 3; Figure 3).

**Figure 4:**
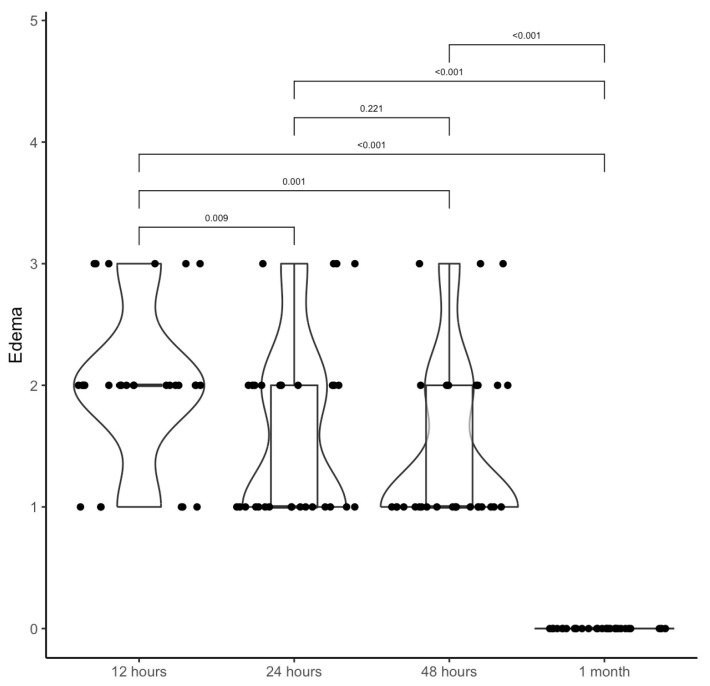
The violin boxplot shows the difference between the Edema score pre-surgery and at 12 h, 24 h, 48 h after surgery, and after 1 month.

**Figure 5:**
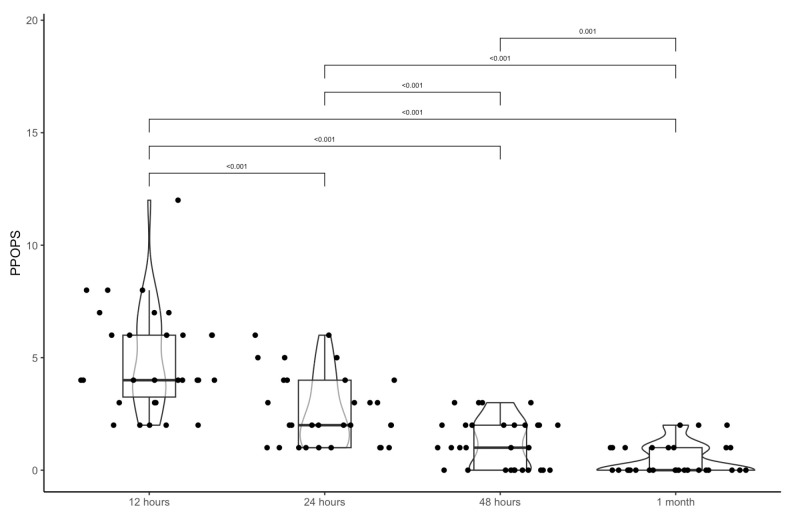
The violin boxplot shows the difference between the PPOPS score pre-surgery and at 12 h, 24 h, and 48 h after surgery, and after 1 month.

The average values of the PPOPS questionnaires were at 12 h, the mean value was 4.9 ± 2.3; at 24 h, the mean value 2.7 ± 1.6; at 48 h, the mean value 1.2 ± 1.1; and at 1 month, the mean value 0.5 ± 0.7 (Table 4). There was a statistical difference between times (Table 4; Figure 4 and Figure 5).

No hemorrhagic complications after tonsillectomy were reported.

In five cases, the suture partially extruded at the palatal level without affecting the procedure’s success. In 2 cases, the extrusion was located on the superior pole of the anterior tonsillar pillar. The patient complained of a foreign body sensation and slight swallowing difficulty. Therefore, in all these cases, the thread was cut without any long-term consequences on the stability of the palate.

## 4. Discussion

Traditionally, ablative surgical techniques (e.g., uvopalatalpharyngoplasty, LAUP) were the treatment for OSA patients. They were based on the erroneous concept that palatal tissue demolition resolved OSA symptomatology [3,4]. However, these techniques had a variety of postoperative complications (e.g., dysphagia, rhinolalia, and velopharyngeal insufficiency) coupled with poor results [17].

Nowadays, our knowledge regarding the anatomy and physiology of OSA highlights the significant role of pharyngeal and palatal collapse, indicating that this structure should be preserved and not ablated.

Barbed palatal surgery has been developed to reduce palatal demolition and promote remodeling and repositioning of the palatal system. In 2014, Vicini et al. proposed BRP and obtained good short—and long-term results [5,6,16]. They used a conservative barbed technique, including three demolition steps: tonsillectomy, fat pad removal, and partial PPM transection [5]. Therefore, BRP involves removing specific tissues to reposition the palate and pharyngeal structures. This technique transcut palatopharyngeal muscle to facilitate repositioning, allowing for better airway patency by securing the soft palate in a more advantageous position.

In 2024, Magliulo et al. proposed a new technique comparing a palatal system with a cable-stayed bridge [7]. BSBP does not remove tissue but instead focuses on stabilizing the existing pharyngeal and palatal structures using barbed sutures. This method creates a “bridge” to support the airway, preventing collapse and maintaining openness without tissue excision. This new surgical procedure, BSBP, is more conservative than BRP since it does not require fat pad removal and partial transection of PPM. Tonsillectomy is unavoidable since exposure to PPM and PPG is like that of barbed palatal surgery.

Schwab has shown that the LPW (lateral pharyngeal wall) plays a part in the pathophysiology of OSAS [18,19]. Therefore, the narrowing of the LPW is the only independent risk factor for OSAS.

In OSA, the link between the pharyngeal muscles is disrupted as the LPW and soft palate collapse and enlarge. The fibrous attachments between these muscles become weak and disordered due to negative pharyngeal airway pressure. Therefore, BSBP aims to restore proper anatomical oropharyngeal space, increasing the system’s elasticity. The transcut of PPM is converted into a barbed longitudinal reinforcement of the muscles. This allows for the restoration of the strength of the muscle, which is rigid but elastic, by internal barbed suture reinforcement and a standard histological structure (no fibrosis due to the cut). At the same time, its position is modified, transposing it from a posterior position to a lateral position. In this way, muscle contraction contributes to opening the oropharyngeal space.

BSBP produces adequate postoperative polygraphic results, with a significant reduction in AHI and ODI maintained even at a one-year follow-up [7].

However, observing how BSBP led to extremely rapid rehabilitation in the first two days after surgery was interesting.

To the best of our knowledge, no specific studies in the literature evaluate or classify immediate postoperative clinical symptoms and edema in a standardized manner. Furthermore, few studies address postoperative discomfort in patients treated with barbed pharyngoplasty [14,15,16,20].

On the contrary, this preliminary study aimed to collect data about patients’ immediate signs and symptoms during the first 48 h and at the one-month follow-up assessment.

Our data demonstrated that PPOPS scores showed a statistically significant difference as early as between 12 and 24 h postoperatively, and this effect was maintained at the 1-month follow-up. Furthermore, the mean values were lower than those reported for other barbed techniques, with a mean value at the 12 h assessment (4.9 ± 2.3). In 2020, Iannella et al. reported a mean value of PPOPS in their study group treated with BRP of 9.57 in 5 years of follow-up [16]. On the other hand, our results align with the data reported by Rashwan et al. [21], who described the mean values of BRP as 2.4, again after 5 years. Our data is probably like that of Rashwan et al. because both studies were based on small samples, but an extensive sample study is underway to corroborate the current data [20,21].

According to other studies, patients gave positive answers to questions 9 and 10, which were solved in the first months after surgery. On the contrary, the BSBP population had no foreign body sensation or sticky mucus in the throat. A possible explanation for this is related to clinical findings (Figure 1): limited edema, especially of the uvula, was visible only during the first 12 h after surgery. Throat pain was moderate, and nutrition was rapidly recommended in the first 12–24 h after surgery. This aspect may also be attributed to the total absence of external sutures on the tonsillar fossa, preventing hemorrhagic complications.

To our knowledge, while postoperative outcomes following barbed pharyngoplasty have been previously reported, there is a lack of studies providing a standardized, quantitative assessment of immediate postoperative symptoms and edema using validated scoring systems such as PPOP and Ezzat score. In this context, our findings highlight a favorable short-term recovery profile characterized by minimal pain and rapid local edema reduction within the first hours and days following surgery.

This is one of the most crucial differences with other techniques, such as BRP. In this latter, at the end of the procedure, the inferior portion of the tonsillectomy bed is opened to allow hemostasis if bleeding occurs [22]. On the contrary, BSBP has a natural internal closure of the tonsillar fossa. In our series, it was not necessary to revise tonsillectomy, and in case of bleeding, revision is also possible, such as cutting between the margins of the tonsillar fossa.

We attribute the absence of bleeding to a minor risk of infection or the mechanical action of food during the postoperative period. Moreover, the lower perception of pain cancels the need to use drugs such as NSAIDs (Non-Steroidal Anti-Inflammatory Drugs) that may increase the risk of bleeding.

Finally, according to the BRP results, BSBP also received a favorable judgment from the patients treated, suggesting that it is a valid method for resolving the OSA problem.

This preliminary study’s limitations include the small sample and the lack of a reliable comparison with other types of palatal surgery and BRP. Therefore, many studies dealing with large samples are underway to confirm previous findings and increase the available data.

## Figures and Tables

**Table 1:** AHI, ODI, and ESS values before surgery and after 6 months. The mean (SD) for each variable in the first row is reported, while the median (IQR) is in the second row.

	Time 0	Time 1	*p*-Value
AHI	23.4 (2.1) 23.4 (21.8; 25.4)	7.2 (2.6) 6.8 (4.9; 8.0)	<0.001
ODI	21.0 (4.1) 21.9 (16.4; 22.8)	6.5 (2.1) 6.2 (5.1; 7.1)	<0.001
ESS	5.1 (3.4) 4 (3; 8)	3.5 (2.5) 3 (1; 6)	<0.001

**Table 2:** VAS at 12 h, 24 h, 48 h, 1 month, and comparison between times. The mean (SD) was reported in the first row, while the median (IQR) was reported in the second row.

12 h	24 h	48 h	1 Month
VAS→3.0 (1.5)	2.5 (1.2)	2.4 (0.9)	0.2 (0.5)
2 (2; 4)	2 (2; 2)	2 (2; 2)	0 (0; 0)
			***p***
	12 h	24 h	0.048
	12 h	48 h	0.045
	12 h	1 month	<0.001
	24 h	48 h	1.000
	24 h	1 month	<0.001
	48 h	1 month	<0.001

**Table 3:** Ezzart score of edema at 12 h, 24 h, 48 h, and 1 month, and comparison between times. The mean (SD) was reported in the first row, while the median (IQR) was reported in the second row.

12 h	24 h	48 h	1 Month
Edema→2.0 (0.6)	1.6 (0.7)	1.4 (0.7)	0.0 (0.0)
2 (2; 2)	1 (1; 2)	1 (1; 2)	0 (0; 0)
			***p***
	12 h	24 h	0.009
	12 h	48 h	0.001
	12 h	1 month	<0.001
	24 h	48 h	0.221
	24 h	1 month	<0.001
	48 h	1 month	<0.001

**Table 4:** PPOPS score at 12 h, 24 h, 48 h, 1 month, and comparison between times. The mean (SD) was reported in the first row, while the median (IQR) was reported in the second row.

12 h	24 h	48 h	1 Month
PPOPS→4.9 (2.3)	2.7 (1.6)	1.2 (1.1)	0.5 (0.7)
4 (3.2; 6)	2 (1; 4)	1 (0; 2)	0 (0; 1)
			***p***
	12 h	24 h	<0.001
	12 h	48 h	<0.001
	12 h	1 month	<0.001
	24 h	48 h	<0.001
	24 h	1 month	<0.001
	48 h	1 month	0.001

## Data Availability

All significant data are reported in the manuscript.

## Supplementary Materials

The following supporting information can be downloaded at: https://www.mdpi.com/article/10.3390/jcm14207402/s1, Video S1: Barbed Stayed Bridge Pharyngoplasty; Video S2: Immediate postoperative clinical evidence.

## Abbreviations

The following abbreviations are used in this manuscript:

OSA	Obstructive Sleep Apnea
CPAP	Continuous Positive Airway Pressure
BRP	Barbed Reposition Pharyngoplasty
BSBP	Barbed Stayed Bridge Pharyngoplasty
PPM	Palatopharyngeal Muscle
PGM	Palatoglossal Muscle
HSAT	Home Sleep Apnea Testing
AHI	Apnea–Hypopnea Index
AASM	American Academy of Sleep Medicine
DISE	Drug-Induced Sleep Endoscopy
VOTE	Velum, Oropharynx, Tongue, Epiglottis (classification system for airway collapse)
IV	Intravenous
VAS	Visual Analog Scale
PPOPS	Palatal Postoperative Problems Score
ODI	Oxygen Desaturation Index
t90%	Percentage of Time with Oxygen Saturation <90%
LPW	Lateral Pharyngeal Wall
OSAS	Obstructive Sleep Apnea Syndrome
LAUP	Laser-Assisted Uvulopalatoplasty
NSAIDs	Non-Steroidal Anti-Inflammatory Drugs
